# Facial profile esthetic preferences: perception in two Brazilian states

**DOI:** 10.1590/2176-9451.20.3.088-095.oar

**Published:** 2015

**Authors:** Marina Detoni Vieira de Oliveira, Bruno Lopes da Silveira, Cláudia Trindade Mattos, Mariana Marquezan

**Affiliations:** 1 Private practice, Passo Fundo, Rio Grande do Sul, Brazil; 2 Adjunct professor, Universidade Federal de Santa Maria (UFSM), School of Dentistry, Santa Maria, Rio Grande do Sul, Brazil; 3 Adjunct professor, Universidade Federal Fluminense (UFF), School of Dentistry, Santa Maria, Rio Grande do Sul, Brazil; 4 Odontologist, Universidade Federal de Santa Maria (UFSM), Santa Maria/RS, Brazil

**Keywords:** Orthodontics, Face, Esthetics

## Abstract

**OBJECTIVE:**

The aim of this study was to assess the regional influence on the perception of facial profile esthetics in Rio de Janeiro state (RJ) and Rio Grande do Sul state (RS), Brazil.

**METHODS:**

Two Caucasian models, a man and a woman, with balanced facial profiles, had their photographs digitally manipulated so as to produce seven different profiles. First year dental students (laypeople) assessed the images and classified them according to their esthetic preference.

**RESULTS:**

The result of the *t* test for independent samples showed differences among states for certain facial profiles. The female photograph identified with the letter 'G' (mandibular retrusion) received higher scores in RS state (p = 0.006). No differences were found for male photographs (p > 0.007). The evaluators' sex seemed not to influence their esthetic perception (p > 0.007). Considering all evaluators together, ANOVA/Tukey's test showed differences among the profiles (p ≤ 0.05) for both male and female photographs. The female photograph that received the highest score was the one identified with the letter 'F' (dentoalveolar bimaxillary retrusion/ straight profile). For the male profiles, photograph identified with the letter 'E' (dentoalveolar bimaxillary protrusion/ straight profile) received the best score.

**CONCLUSION:**

Regional differences were observed regarding preferences of facial profile esthetics. In Rio de Janeiro state, more prominent lips were preferred while in Rio Grande do Sul state, profiles with straight lips were favored. Class III profiles were considered less attractive.

## Abstract (pt)

**OBJETIVO:**

o objetivo desse estudo foi avaliar a influência regional na percepção da estética do perfil facial nos estados do Rio de Janeiro (RJ) e Rio Grande do Sul (RS), Brasil.

**MÉTODOS:**

dois modelos leucodermas, um homem e uma mulher, com perfis faciais harmoniosos, tiveram suas fotografias de perfil editadas de modo a produzir sete perfis diferentes. As imagens foram avaliadas por estudantes do primeiro ano do curso de Odontologia (leigos), que as classificaram quanto à sua preferência estética.

**RESULTADOS:**

o resultado do teste *t* para amostras independentes demonstrou haver diferença entre as regiões, quanto à preferência estética de determinados perfis faciais. A fotografia feminina nomeada com a letra 'G' (retrusão da mandíbula) recebeu os maiores escores em RS (p = 0,006). Para as fotografias masculinas, não houve diferença (p > 0,007). O sexo dos avaliadores não exerceu influência na percepção da estética do perfil (p > 0,007). Considerando todos os avaliadores em conjunto, o teste ANOVA/Tukey mostrou haver diferença entre os perfis avaliados (p ≤ 0,05) tanto para as fotografias femininas quanto para as masculinas. A fotografia feminina preferida foi a com a letra 'F' (biretrusão dentoalveolar/perfil reto). A fotografia masculina preferida foi a 'E' (biprotrusão dentoalveolar/perfil reto).

**CONCLUSÃO:**

foi observada diferença regional quanto à preferência estética do perfil. No estado do Rio de Janeiro, houve preferência por lábios mais proeminentes; enquanto no estado do Rio Grande do Sul, os perfis considerados mais atrativos foram os retos. Os perfis considerados menos atrativos foram os que correspondem à Classe III.

## INTRODUCTION

Facial esthetics is a physical trait pursued in society. People with balanced facial characteristics are supposedly considered as more competent, better succeeded and happier. Adults and children with attractive faces are judged favorably and treated more positively when compared to the least attractive ones.^1^

Beauty has hence become object of many studies for a wide range of professionals, such as estheticians, plastic surgeons and dental surgeons, including orthodontists. Orthodontics plays an important role in facial esthetics due to the positioning of anterior teeth and the strong influence it bears on overlying lips.^2,3^

Facial profile harmony and balance can be measured, and representative values of a standard profile can be reached.^4^^-^ 10 However, it is well known that this standard can vary as a result of interracial marriages^2^and that orthodontic treatment should take into account individual and racial^11^^-^ 15 characteristics, as well as the individual's personal concept of beauty.

Orthodontic patients have different backgrounds, with varying ancestors, levels of instruction, social status, gender and perception of beauty. Under such perspective, the orthodontic science must adapt its concepts and norms so that a standard outcome of treatment is avoided. The orthodontist must realize that the patient is unique, and that self-esteem after treatment conclusion is as important as technical outcomes. This manuscript aimed at assessing the esthetic preferences in perception of male and female profiles in two Brazilian states - Rio Grande do Sul and Rio de Janeiro; verifying whether profile perception differs between men and women evaluators; and determining which profiles are favored by the population. Rio de Janeiro bears a strong African influence whereas Rio Grande do Sul is characterized by European influence. This study will help professionals understand and better achieve esthetic expectations of patients undergoing orthodontic treatment within different parts of Brazil.

## MATERIAL AND METHODS

**Figure 1.**
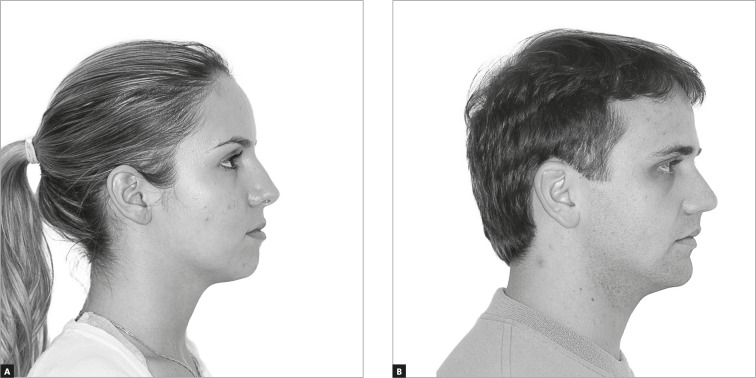
Models selected for s profile photographs: A) female; B) male.

This research was submitted and approved by Faculdade Ingá (UNINGÁ) Institutional Review Board. Two Caucasian models, one male and one female (Fig 1), with harmonic profiles and skeletal and dental Class I relationship(confirmed by Steiner's lateral cephalometric tracings) were chosen for facial profile preference evaluation. Their ages ranged from 20 to 25 years, both had a pleasant profile and lack of apparent sagittal discrepancies. The original profiles were not precisely straight according to Steiner's S-line because it was difficult to find models with upper and lower lips exactly touching the S-line, which is drawn from the soft tissue pogonion to the midpoint of the columella of the nose. However, the models selected for this study were very close to it. The female model had slight protrusion while the male model had slight retrusion.

Both models were informed of the aims of the study and signed an informed consent form agreeing to participate. Specific orthodontic records comprised a profile photograph (in natural head position) and a lateral cephalogram, both of which were taken at a dental radiology laboratory by a trained specialist. The camera used was a Canon EOS Rebel XT (Canon^(r)^ Macro Lens EF 100mm 1:2.8 USM, Tokyo, Japan) initially in color scale and dimensions of 1664 x 2496 pixels. Black and white photographs were later used so that the color of the skin, eyes and hair would not influence evaluation.^16,17^ The x-ray was taken using Gendex Orthoralix 9200 (Milan, Italy) and its dimensions were of 1384 x 1922 pixels.

The initial tracing of each lateral cephalogram comprised SN-line, Steiner's S-line and a vertical reference line (VRL) drawn perpendicular to SN -7^o^ (line starting at S with a 7^o^ clockwise difference from the SN-line).^18^^,^ 19 ^,^ 20

**Figure 2.**
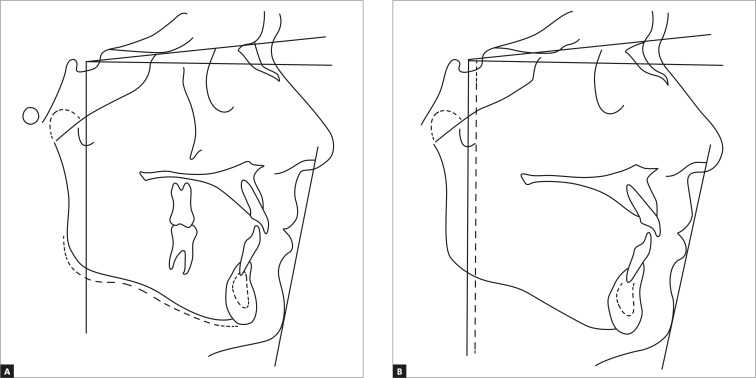
Sample tracing used to guide profile changes: A) original male tracing; B) modified male tracing (bimaxillary protrusion: dotted line corresponds to a 3-mm displacement of the VRL).

Based on the original tracings, six new tracings were produced for each model, male and female, simulating changes in facial profile, so that the alveolar portions of the maxilla and/or mandible were moved 3 mm forward from the VRL, simulating protrusion; or 3 mm backward from the VRL, simulating retrusion (Fig 2). Tracings were manipulated as follows:

1. Bimaxillary protrusion: 3 mm forward positioning of the alveolar segments of the maxilla and mandible from the VRL, producing upper and lower labial protrusion without altering the position of the basal bones.
2. Mandibular protrusion: 3 mm forward positioning of the mandible from the VRL.
3. Mandibular retrusion: 3 mm backward positioning of the mandible from the VRL.
4. Maxillary retrusion: 3 mm backward positioning of the maxilla from the VRL.
5. Maxillary protrusion: 3 mm forward positioning of the maxilla from the VRL.
6. Bimaxillary retrusion: 3 mm backward positioning of the alveolar segments of the maxilla and mandible from the VRL, producing upper and lower labial retrusion without altering the position of the basal bones.

The original photographs were digitally manipulated by a graphic designer using Adobe Photoshop CS5 Extended software (version 12.1x64 Copyright 1990-2011^(r)^, Dublin, Ireland) generating six new images for each model. The original images underwent basic leveling of brightness and contrast. The predictive tracings were scanned one at a time overlying the original photograph, to guide changes made to the image. The tracing layer was removed and the modified image was saved.

**Figure 3.**
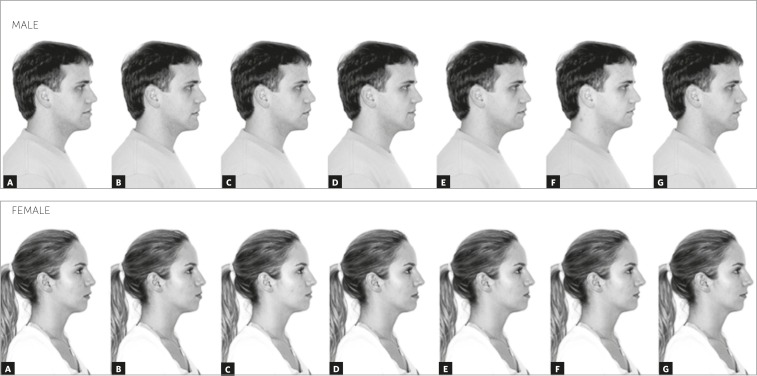
Modified photographs randomly distributed.

New images were sorted according to the model's sex and were randomly presented as indicated by the designer (Fig 3). Photographs were registered as follows:

**1) Males**
A = Mandibular protrusion;
B = Bimaxillary retrusion;
C = Maxillary protrusion;
D = Maxillary retrusion;
E = Bimaxillary protrusion;
F = Original photograph;
G = Mandibular retrusion.

**2) Females**
A = Maxillary protrusion;
B = Bimaxillary protrusion;
C = Original photograph;
D = Maxillary retrusion;
E = Mandibular protrusion;
F = Bimaxillary retrusion;
G = Mandibular retrusion.

Photographs were printed in white couche A3 paper (420 mm x 297 mm).

The evaluators were first year undergraduates enrolled at the School of Dentistry of Universidade Federal do Rio de Janeiro (UFRJ), Rio de Janeiro (RJ); and at the School of Dentistry of Faculdade Meridional IMED, Passo Fundo (RS). They were selected for having little or no technical knowledge of facial profiles, and were considered as laypeople because when tests were applied they had not yet covered the disciplines that emphasize profile esthetics, such as Orthodontics or Orofacial Surgery. Previously to photographic evaluation, permission was required from the directors of the participating institutions, who signed a term agreeing to have the study conducted in the aforementioned institutions. Undergraduates also signed an Informed Consent Form agreeing to participate in the study. Evaluation was carried out individually so that one evaluator would not influence the other. Each evaluator received cards with grouped photographs: one with all seven female photographs, the other with all seven male photographs (Fig 3), and an instruction card for facial profile evaluation.

Evaluators gave scores ranging from 1 to 7 for each group of photographs without repeating the score. Number 7 represented the most balanced, pleasant and beautiful profile; and number 1 represented the least balanced, unpleasant, and ugly one. This card also contained questions regarding evaluator's sex, age, place of birth and current city.

After collection, data were tabulated and statistically analyzed by the Statistical Package for Social Sciences software (version 18, SPSS Inc., Chicago, Illinois, USA). Considering evaluators as two distinct groups, according to Brazilian evaluated states, scores attributed by the participants were compared by means of unpaired t-test. Considering evaluators as two groups according to sex, attributed scores were also compared by t-test. Bonferroni correction was applied and the level of significance for the *t* test was set at 0.007. Considering all evaluators as one group, the differences in scores attributed to each one of the seven photographs was evaluated by analysis of variance (ANOVA) followed by Tukey's multiple comparisons test.

## RESULTS

Twenty-nine first year Dental School undergraduates born and residing in Rio Grande do Sul, and thirty-six undergraduates born and residing in Rio de Janeiro filled out the research form. Their ages ranged from 17 to 23.

The *t* test for independent samples detected a difference between states when it came to esthetic preference of certain facial profiles. Female photograph 'G' received the highest scores in Rio Grande do Sul (p = 0.006) (Table 1). No difference was found in male photograph evaluation (p > 0.007) (Table 2).

**Table 1.** Mean and standard deviation of scores attributed for female photographs and result of statistical tests.

Photograph	Total sample Mean ± SD	RS sample Mean ± SD	RJ sample Mean ± SD	RS x RJ p-valor (t-Student)
A	3.64 ± 1.56 (c)	3.17± 1.73	4.03 ± 1.92	0.45
B	4.35 ± 1.52 (c,d)	4.31 ± 1.31	4.39 ± 1.49	0.24
C	4.46 ± 1.83 (d)	4.24 ± 1.35	4.64 ± 1.33	0.89
D	2.72 ± 1.89 (b)	2.59 ± 1.05	2.83 ± 1.57	0.02
E	1.61 ± 2.11 (a)	1.69 ± 1.16	1.56 ± 0.73	0.06
F	6.01 ± 2.21 (f)	6.10 ± 1.39	5.94 ± 1.54	0.40
G	5.23 ± 1.94 (e)	5.90 ± 1.11	4.69 ± 1.68	0.006*

RS= Rio Grande do Sul state; RJ= Rio de Janeiro state. Different letters indicate statistically significant difference for ANOVA/Tukey test (p = 0.05). *Indicate statistical significant difference for t-test (p = 0.007).

**Table 2.** Mean and standard deviation (SD) of scores attributed for male photographs and result of statistical tests.

Photograph	Total sample Mean ± SD	RS sample Mean ± SD	RJ sample Mean ± SD	RS x RJ p-valor (t-Student)
A	1.83 ±1.89 (a)	1.66 ± 1.17	1.97 ± 1.59	0.09
B	4.07 ± 1.96 (c)	3.93 ± 1.53	4.19 ± 1.52	0.58
C	4.18 ± 1.91 (c)	4.17 ± 1.58	4.19 ± 1.60	0.69
D	2.73 ± 1.83 (b)	2.86 ± 1.84	2.16 ± 1.41	0.34
E	5.96 ± 2.02 (e)	5.59 ± 1.59	6.28 ± 1.38	0.31
F	5.09 ± 1.77 (d)	5.41 ± 1.40	4.83 ± 1.40	0.50
G	4.00 ± 1.28 (c)	4.14 ± 1.70	3.89 ± 1.78	0.45

RS= Rio Grande do Sul state; RJ= Rio de Janeiro state. Different letters indicate statistically significant difference for ANOVA/Tukey test (p = 0.05). *Indicate statistical significant difference for t-test (p = 0.007).

*T* test for independent samples did not detect any differences (P > 0.007) when verifying whether evaluators' sex influenced profile analysis.

Considering all evaluators as one group, ANOVA/Tukey's tests showed differences in all seven photographs evaluated by both males and females(P < 0.001). For the female photographs, preference was for photograph 'F', followed by photograph 'G', then by photographs 'C' and 'B', then by 'A' and 'D', and finally by 'E' (Table 1). For male photographs, preference was for photograph 'E', followed by photograph 'F', then by photographs 'C', 'B' and 'G' (no difference between them), photograph 'D', and finally by photograph 'A' (Table 2).

## DISCUSSION

When all evaluators were considered as a single group, the favored profiles were straight, exactly as described by Steiner (lips touching the S-line). These profiles correspond to female photograph 'F' (simulated bimaxillary retrusion in a slightly protrusive model) and male photograph 'E' (simulated bimaxillary protrusion in a slightly retrusive model). Profiles considered the least attractive were the ones corresponding to Class III, namely female photographs 'D' and 'E', and male photographs 'D' and 'A'. These findings support previous studies that state the preference for straight profiles.^21^^-^ 25 According to Johnston et al,^24^ attractiveness reduces gradually as values of SNB increase or decrease by 5^o^. The least attractive profile was considered to be the mandibular protrusion, as found by previous studies.^23,26^

As the male model had slightly retrusive lips with regard to the S line, when the photograph was edited to simulate a bimaxillary protrusion, his profile became straight and was preferred by the evaluators. For the female model, photograph 'B', which corresponded to bimaxillary protrusion, was less appreciated than the closer-to-straight profiles seen in photographs 'F', 'G' and 'C'. This result differs from the current orthodontic trend that believes more protrusive profiles are preferred. However, it is worth remembering that the chin and nose will continue to grow forward during adult life,^20,27^ tending to produce a concave profile (the least acceptable according to the results). Thus, it seems wise to end patient treatment with slightly convex profiles.

Minor differences were noted between average scores attributed to the profiles, in the states of Rio Grande do Sul and Rio de Janeiro. As a general rule, more prominent lips in the female profile were better appreciated by Rio de Janeiro evaluators than those in Rio Grande do Sul (photographs 'A', 'B', 'C'). Closer-to-straight profiles were preferred by evaluators in Rio Grande do Sul (photographs 'F' and 'G'). However, only one profile score demonstrated statistical difference. Photograph 'G', showing mandibular retrusion and a straight profile, received higher scores in Rio Grande do Sul (p = 0.006) (Table 1). Racial and cultural influences seem to explain such results. Rio Grande do Sul was colonized by Europeans (81.5% of the population is of European descendants) with great Italian and German influence, displaying straight profiles more often than African populations.^15^ On the other hand, Rio de Janeiro has an expressive African background (32% of the population is of African descendants);^28^ hence, more protrusive profiles are observed.^15^ According to Oliveira Jr,^29^ in Brazil, cephalometric values change from region to region, since Brazil is a country with continental dimensions inhabited by a diverse ethnic, cultural and religious population, in contrast to other nations. From a genetic standpoint, the mixing of races in Brazil accentuates the difficulty in finding cephalometric measurements capable of epitomizing a Brazilian pattern.

In this context, the literature has related statistically significant differences in linear and angular standard values between Caucasian and African populations.^15^ In the latter, the structure is larger; incisor tipping and protrusion are more accentuated; position of the maxilla, length of the mandible and location of the porion are different from Caucasians. In addition, black and white laypeople and professionals display different esthetic preferences.^30^ If a certain region has larger African influence, the preferences of their locals seem to be different.

As for male profiles, none displayed statistically significant differences when state scores were compared. Despite more protrusive profiles (photograph 'E') receiving higher scores in Rio de Janeiro, the bimaxillary retrusive profile (photograph 'B') was also preferred in that state. In Rio Grande do Sul, however, more retrusive profiles obtained higher scores ('F', original, slightly retrusive; 'G', mandibular retrusion; 'D', maxillary retrusion).

As for evaluators' sex, no statistically significant difference was detected in the scores attributed. Men and women had similar perception when it came to female and male profiles. This result differs from data found in other studies, which concluded that sex bears influence on esthetic preferences.^22,25^

A strong point in this study is the method chosen, similar to that proposed by Mantzikos,^21^ Turkkahraman and Gokalp,^22^ Soh,^23^ and Cala;^25^ however, in the present study, images were not altered by the software used to predict orthognathic surgery results, as other studies did. In order to create anterior and posterior changes, the original cephalometric tracing was modified according to the method proposed by Stephens,^19^ Erdinc et al,^18^ and Mattos et al^20^ in which a vertical reference line is drawn perpendicular to another line, drawn 7 degrees clockwise from line SN (SN -7^o)^. Taking this line perpendicular to the x-axis as reference, protrusive and retrusive changes of 3 mm were carried out in the maxilla, mandible or both.

The number of evaluators was a limitation. Sample size could have been larger, as it was in the studies of reference, varying from 92^23^ to 2.651.^21^ Even with a smaller sample (n = 65) in comparison to similar studies, it was possible to detect statistically significant differences in some of the profiles evaluated.

There are yet many factors that can influence facial profile preferences in the various parts of Brazil, such as skin color, hair, eyes, culture, geographic location, level of instruction and social-economic status. Studies investigating the heterogeneity of the facial profile not only in Caucasians, but in African descendants, Native Americans, and in other racially mixed populations are necessary. Studies are still anticipated regarding facial esthetic preferences in different regions of Brazil.

## CONCLUSIONS

More prominent lips are preferred in Rio de Janeiro state while in Rio Grande do Sul state profiles with straight lips were favored. However, only the preference for profiles with mandibular retrusion and straight profile in Rio Grande do Sul showed statistically significant difference.

As for the evaluators' sex, profile perception was not influenced by this variable.

Considering all evaluators as one group, straight profiles were preferred and those reflecting Class III relationship were considered the least attractive.
